# Processing Facial Expressions of Emotion: Upright vs. Inverted Images

**DOI:** 10.3389/fpsyg.2013.00054

**Published:** 2013-02-14

**Authors:** David L. Bimler, Slawomir J. Skwarek, Galina V. Paramei

**Affiliations:** ^1^School of Arts, Development and Health Education, Massey UniversityPalmerston North, New Zealand; ^2^Institute for Leadership and Personal Management, University of St. GallenSt. Gallen, Switzerland; ^3^Department of Psychology, Liverpool Hope UniversityLiverpool, UK

**Keywords:** facial expressions, emotion, inversion, Same/Different, signal detection theory, multidimensional scaling, categorization, featural analysis

## Abstract

We studied discrimination of briefly presented upright vs. inverted emotional facial expressions (FEs), hypothesizing that inversion would impair emotion decoding by disrupting holistic FE processing. Stimuli were photographs of seven emotion prototypes, of a male and female poser (Ekman and Friesen, 1976), and eight intermediate morphs in each set. Subjects made speeded *Same*/*Different* judgments of emotional content for all upright (U) or inverted (I) pairs of FEs, presented for 500 ms, 100 times each pair. Signal Detection Theory revealed the sensitivity measure *d*′ to be slightly but significantly higher for the upright FEs. In further analysis using multidimensional scaling (MDS), percentages of *Same* judgments were taken as an index of pairwise perceptual similarity, separately for U and I presentation mode. The outcome was a 4D “emotion expression space,” with FEs represented as points and the dimensions identified as *Happy–Sad*, *Surprise*/*Fear*, *Disgust*, and *Anger*. The solutions for U and I FEs were compared by means of cophenetic and canonical correlation, Procrustes analysis, and weighted-Euclidean analysis of individual differences. Differences in discrimination produced by inverting FE stimuli were found to be small and manifested as minor changes in the MDS structure or weights of the dimensions. Solutions differed substantially more between the two posers, however. Notably, for stimuli containing elements of *Happiness* (whether U or I), the MDS structure showed signs of implicit categorization, indicating that mouth curvature – the dominant feature conveying *Happiness* – is visually salient and receives early processing. The findings suggest that for briefly presented FEs, *Same*/*Different* decisions are dominated by low-level visual analysis of abstract patterns of lightness and edge filters, but also reflect emerging featural analysis. These analyses, insensitive to face orientation, enable initial positive/negative Valence categorization of FEs.

## Introduction

Facial expressions (FEs) contain information about emotional state, but despite decades of research, the nature of this information is still far from definite. Nor is it clear what stages of visual processing are involved in perception of a facial expression (FE), i.e., how face pictorial cues conveying this information are translated into a mental/affective representation. If some kind of perturbation or degradation of the stimuli selectively disrupted some aspects of facial information more than others, this would provide clues to the underlying mechanics of FE perception.

A research tradition has examined the effects of *inverting* FE stimuli as a simple way of disrupting their perception. An extreme possibility is that inverting FEs takes away their emotional content (e.g., Parks et al., 1985). However, examining misidentifications among inverted FEs displayed for 15 s, McKelvie (1995) found that although these were mislabeled more often than upright FEs, they still conveyed emotions more accurately than chance would predict. The overall pattern of confusions was similar in both presentation modes, with relatively high confusion rates between particular pairs of emotions (e.g., *Fear* misread as *Surprise* and vice versa). This finding has since been replicated with briefer and with unlimited exposures (Prkachin, 2003; Calvo and Nummenmaa, 2008; Derntl et al., 2009; Narme et al., 2011). It indicates that the disruptive impact of inversion upon FE processing is not complete, and is general rather than being confined to specific expressions.

The approach taken here is to collect and analyze perceptual similarities, a well-established methodology in the FE domain. In the studies cited above, misidentifications of displayed emotions can be regarded as a behavioral index of similarity between presented FE stimuli and implicit, latent prototypes of emotional expression. Elsewhere the data consist of explicitly judged inter-stimulus similarities (e.g., Gladstones, 1962; Stringer, 1967; Bimler and Kirkland, 2001). Here the similarities take the form of another behavioral measure: the *probability* of wrongly identifying two (similar) FE stimuli as *duplicates* in a speeded *Same*/*Different* (*S*/*D*) task. *S*/*D* errors have been used as a surrogate for similarity in several studies of FE categorical perception, in order to locate the boundary between two emotion categories (e.g., Calder et al., 1996, Experiment 4; Roberson et al., 1999)^1^. Our analysis focuses on a comparison between the overall structure of similarities among inverted FEs and the similarities among the same stimuli when they are upright. The comparison uses several complementary metrics to quantify any upright/inverted difference.

The challenge in the analysis of similarities is to extract any clues the raw data might contain about perceptual processing, and in particular, about the *level* of processing accessed when determining similarity. In themselves, similarity data do not speak directly to the presence of emotional content after inversion, but any evidence that the stimuli are perceived categorically could be taken as a sign of the presence of emotional categories.

At one extreme, subjects might base their FE comparisons upon the visual images in a relatively raw form, i.e., derivative-of-Gaussian edge filters (Marr, 1982; Dailey et al., 2002), where inversion would have little impact. Conversely, later stages of visual processing would be involved if the comparisons draw upon cues or information *extracted* from FE stimuli: in particular, featural and/or configural information^2^. If both forms of information are extracted, and one is more accessible when faces are upright, then inverting the stimuli will reduce the contribution of these cues to perceived inter-stimulus dissimilarity.

There is evidence from a closely related perceptual domain – that of facial *identity* and recognition – that inversion selectively disrupts configural (second-order) cues. Inversion impairs face recognition in a qualitative rather than quantitative way (Yin, 1969; Leder and Bruce, 2000). In comparison, analysis of featural information is disrupted to a lesser degree (Farah et al., 1995; Maurer et al., 2002). The question, then, is whether configural cues are equally important in processing FEs.

The *configural* aspect of a face consists of how its features are arranged; it is synonymous with “relational” or “spatial-relational” (Diamond and Carey, 1986; Bartlett and Searcy, 1993). Conversely, the featural aspect consists of local descriptions, or feature-specific cues to emotional state, e.g., mouth curvature, eye openness, or eyebrow lowering.

It has been argued that “the various expressions reflect spatial relationships and distinctive features to different degrees” (McKelvie, 1995, p. 332). If so, an empirical answer is possible because disruption of configural cues will selectively impact on the transmission of some emotions more than others (Smith and Schyns, 2009). *Disgust* is arguably a configural, “holistic” expression, since local featural correlates for it are elusive (Paramei and Benson, 1998; Bimler and Paramei, 2006). In comparison, *Happiness* and *Anger* are “featural,” relatively “localized” emotions, expressed by the lower and upper half of a FE respectively, whereas *Surprise* is expressed with equal force by both halves (Bassili, 1979; Calder et al., 2000; Bimler and Paramei, 2006; Fiorentini and Viviani, 2009) or the lower face (Smith and Schyns, 2009).

In exploring similarities among upright and inverted FEs, the present study follows a number of precedents by representing FEs as points in a spatial model – a multidimensional “map” – so that geometrical distances between points summarize and approximate the corresponding (dis-)similarities. The map’s dimensions are identified as the affective continuous qualities on which an expression can vary. The algorithms for this form of analysis come under the rubric of multidimensional scaling (MDS). To address the putative effect of image inversion on the extraction of configural cues, we use the particular form of MDS, weighted-Euclidean *individual differences* MDS (Kruskal and Wish, 1978).

This approach attempts to account for multiple sets of similarities by assuming that they all arise from a single “map,” while allowing the *salience* (weight) of some dimensions to vary according to conditions (here: upright vs. inverted), so as to optimize the match between reconstructed inter-stimulus distances and the corresponding dissimilarities in each condition. If inversion were to selectively reduce the salience of one specific dimension, this could be accommodated by compressing the map, reducing the distance between pairs of stimuli that are separated along that direction. A negative finding, i.e., no difference in FE space from inverting the stimuli, would not prove that similarity judgments are determined by featural cues in isolation. As already noted, the judgments may use information from an earlier stage of visual processing.

The spatial metaphor for FEs is only an approximation; an alternative account uses a metaphor of discrete clusters instead (e.g., Calder et al., 1996). It has been argued, though, that both forms of structure may co-exist in FE-similarity data (Dailey et al., 2002; Bimler and Paramei, 2006), much as color perception displays categorical structure as well as a dimensional “color space.” Categorical perception (CP) for FEs is well-attested (e.g., Ekman and Friesen, 1976; Etcoff and Magee, 1992; Calder et al., 1996; Young et al., 1997; Bimler and Kirkland, 2001; Bimler and Paramei, 2006), with the effect of partitioning FE space into categories of prototypical FE of emotion such as of *Surprise*, *Happiness*, etcetera.

The possibility of CP can be explored here because the stimuli include, along with prototypical FEs, ambiguous, intermediate expressions produced by image-interpolation. Thus it is of interest whether the present data exhibit any signs of *categorical* processing for upright and inverted FEs. To the extent that the data show CP for upright or inverted stimuli, one could argue that perceptual processes have implicitly reflected the emotional content of the stimuli, enough to classify FEs by category. We should be prepared, however, for the possibility that the present conditions of brief stimulus exposure and speeded decisions will negate CP for both orientations.

In general, CP can be considered as a non-constant (sigmoidal) relationship between the objective *physical* dissimilarity between stimulus pairs and the corresponding perceptual dissimilarity. The physical interval between two stimuli can be small yet produce a disproportionately large dissimilarity if they straddle a category boundary. The latter is estimated using morphed stimuli interpolated at regular intervals along a perceptual continuum, between emotion prototypes of (for instance) *Happy* and *Angry*. Generally a category boundary emerges somewhere along the gradient. Discrimination tasks, ABX (e.g., Calder et al., 1996; de Gelder et al., 1997; Roberson and Davidoff, 2000) or XAB (e.g., Roberson and Davidoff, 2000), using morphed FEs showed that the error rate and the RTs – two measures of similarity between adjacent stimuli – both dip sharply for some interval along the gradient, coinciding with a transition in the verbal labels assigned to the morphs, which might be consistently identified as *Happy* on one side of the transition and as *Angry* on the other. Other studies used the *S*/*D* task with adults (e.g., Calder et al., 1996, Experiment 4; Roberson et al., 1999, Section 5.2; Shibui et al., 2001) or, with 7-month-old infants, a novelty-preference procedure (Kotsoni et al., 2001), and obtained similar results. It appears that all these tasks are tapping into the high-level processes required to extract *affective* categories from the stimuli.

Retention of CP in inverted FEs is currently controversial. Inversion appears to weaken or remove *category boundaries* along continua of morphed stimuli [de Gelder et al., 1997, Experiment 2); Roberson et al., 1999, Section 5.2)]. However, it has no effect when an emotion *category* is identified and prototypical FEs are employed [*S*/*D* in a visual search task (Lipp et al., 2009); identification of a FE as “happy” or “not happy” (Calvo et al., 2012)].

Categorization, or high-level processing extracting semantic aspects of face perception, is argued to draw upon configural information (de Gelder et al., 1997). More recently, however, it was proposed that categorical processing can be feature-based – for *Happy* expressions with the salient mouth curvature feature – and, thus, precede affective attribution at the stage of configural processing (Calvo et al., 2012).

One can hence expect FE inversion to obviate any effect of CP, in contrast to featural cues with their relative insensitivity to orientation (reviewed by Maurer et al., 2002). The presence or absence of an effect from inversion, therefore, is a test of the proposition that configural content plays a dominant role in decoding emotional content of FEs.

By comparing performance for upright and inverted FEs in the *S*/*D* task, we hope to gain insight into the cognitive mechanics of “low-level”/“high-level” processing, i.e., into “[the] cognitive or functional primitives underlying the perception of FE” that operate at “the locus of emotion perception in the cognitive architecture of the organism” (de Gelder et al., 1997, p. 20).

## Materials and Methods

### Subjects

Two male and two female undergraduate psychology students, aged 21–25 years old, were paid to participate in 30 1-h-long sessions. Informed consent was obtained from all subjects. Gender of FE poser and subject gender were counterbalanced: stimuli from the “MO series” (female poser) were presented to one female subject (DK) and one male (HK); likewise, the “WF series” (male poser) was presented to one female subject (SB) and one male (BF).

### Stimuli

Fourteen monochrome photographs of emotional expressions were selected from *Pictures of Facial Affect* (Ekman and Friesen, 1976). Ekman and Friesen deemed these 14 images to be good examples of seven universal emotion categories (*Happiness*, *Surprise*, *Anger*, *Sadness*, *Fear*, *Disgust*, *Neutral*), as evinced by high accuracy of labeling. Seven images featured a female poser MO while the other featured a male poser WF.

**Figure 1:**
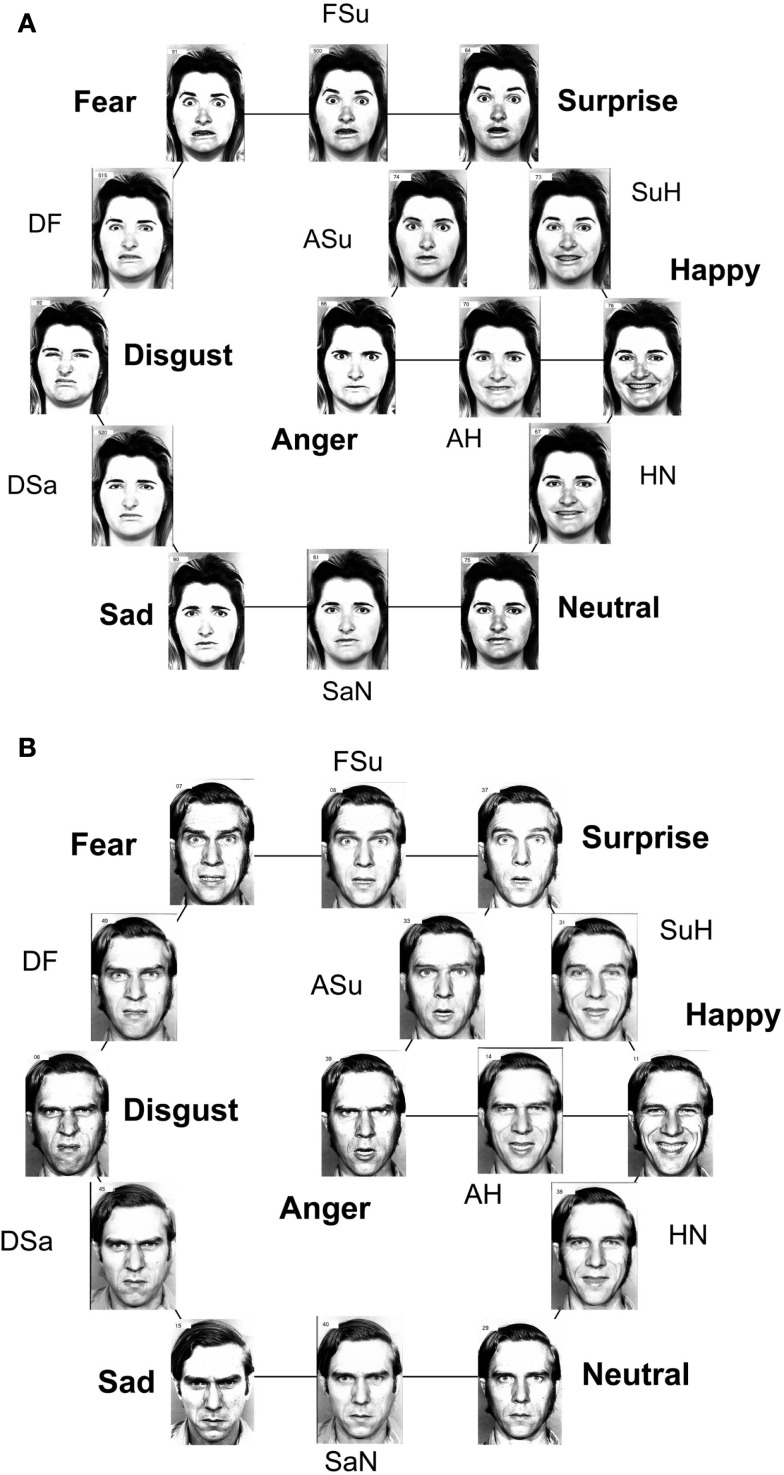
**FE stimuli, arranged in a distorted circumplex**. The prototype FEs are accompanied by emotion names, while abbreviations accompany the intermediate morphs, e.g., *SuH* to indicate the morph between the *Surprise* and *Happiness* prototypes. (Note: circumplex arrangement is chosen for convenience, and not to indicate the similarity relationships among the seven emotional prototypes). **(A)** MO series; **(B)** WF series.

The “MO series” and “WF series” were both extended by using image-interpolation software (Design Studio) to create eight intermediate stimuli, each lying midway along the continuum defined by two emotion exemplars as end-points. Briefly, the “morphing” process involves locating “landmarks” within each prototype. Each triangle defined by three adjacent landmarks in one face can then be transformed smoothly into its counterpart into the other face, allowing stages along the transformation to be interpolated (e.g., Calder et al., 1996; Young et al., 1997). Figure 1 shows the stimuli of both series, where for clarity they have been arranged in a distorted version of the circumplex model (Russell, 1980).

These digitalized stimuli were presented on a 19″ CRT-monitor (V7 N110s), where each image occupied 12.8 × 8.7 cm (subtending 10° × 6.7° at a viewing distance of 74 cm). Measured with a LMT L1009 Luminance Meter, image luminance ranged from 0.23 to 82 cd/m^2^. Ambient lighting in the test room was in the mesopic range (around 10 cd/m^2^).

### Procedure

Each trial consisted of the simultaneous presentation of two FE stimuli, symmetrically side-by-side on the screen with a 3.8 cm gap between them (subtending 3°). After 500 ms, the screen went blank until the subject responded via a two-button keyboard, by pressing the right button “if the two emotions are the *Same*” or the left button if the stimuli were *Different*. Subjects were instructed to respond as quickly and correctly as possible, and to press the buttons with their right and left index fingers respectively. A MS-DOS program (running on a Windows-98 PC) controlled presentations and recorded the response, *Same* or *Different* (as well as RT, from the appearance of the FE pair to the response). Each response was followed by an inter-stimulus interval of 300–400 ms, while a small red fixation cross was displayed on the monitor.

In a single run, all possible 15 × 15 = 225 pairings of FEs were presented in randomized order. Note that 15 of these pairs were indeed identical. Within a single run, all pairs were either upright (U) or inverted (I). Runs alternated between upright and inverted stimulus pairs. For each subject the experiment began with a practice session of one run in each of the U and I modes. There followed ten sessions containing six runs and 20 containing seven runs, totaling to 100 runs with upright pairs and 100 in the inverted mode. These 30 sessions were spread over 4 months including a 2-month break.

Subjects were not led to expect a low proportion of same-stimulus pairs or a high proportion either. Their instructions included no indication of what proportion to expect.

### Analysis

#### Signal detection theory

Signal Detection Theory (SDT) has been applied previously to analyze FE discrimination (Prkachin, 2003; Maxwell and Davidson, 2004; Milders et al., 2008; Smith and Schyns, 2009; Narme et al., 2011). Here we used SDT to analyze the correct recognition of 15 identical pairs and the erroneous *Same* responses to 210 different-expression pairs. For each subject, SDT yielded the sensitivity measure *d*′ and the response criterion *C* (Green and Swets, 1966) for each of the 100 runs, allowing these indices to be plotted against the course of data collection.

To adumbrate the Section “*Results*,” we remark here that most different-expression pairs received a sufficiently high percentage of *Same* responses to rise above the statistical shot-noise. In a preliminary analysis, these “%*Same*” values were examined for any obvious systematic dependence on presentation mode, i.e., whether inter-stimulus differences were more or less evident in U than in I mode.

#### MDS: comparison of solutions for upright vs. inverted facial expressions

If *A* and *B* are two 15-by-15 stimulus matrices of %*Same* values for a given observer and presentation mode, a simple index of similarity between them is *r* ***_AB_*** (the bivariate correlation between corresponding entries in *A* and *B* across all 225 pairs of FEs). An 8-by-8 (4 subjects × 2 presentation modes) table of *r*_AB_ for all pairs of *A* and *B* was examined, to search for obvious effects of inversion and to identify those matrices which exhibit a similar underlying structure.

We went on to treat these %*Same* values as estimates of the similarity between pairs of stimuli (cf. Calder et al., 1996, Experiment 4; Roberson et al., 1999), and to analyze each similarity matrix with non-metric MDS to represent and summarize its structure. The PROXSCAL MDS algorithm (implemented within SPSS) was used for this purpose.

The data proved to be sufficiently robust that separate MDS solutions could be fitted to each %*Same* matrix, i.e., to each combination of subject, poser, and presentation mode. To provide a common framework for qualifying the impact of inversion on perceptions of similarity, U and I mode data were examined separately for each stimulus set, using the “repeated measures” feature of MDS to combine the %*Same* matrices from two subjects into a single configuration. Pooling data also increases the accuracy of the solutions. This led to four MDS solutions, which can be labeled MO-U, MO-I, WF-U, and WF-I.

We quantified the concordance between pairs of MDS solutions, U vs. I, in three complementary ways to avoid the limitations of any single metric for comparison.

(a) *Cophenetic correlation*. This is the correlation (*c*) between each inter-point distance in the U solution and its counterpart in the I solution;
(b) *Procrustes analysis* consists of superimposing the U and I solutions, rotating and rescaling them to minimize the total distance (*g_l_*) between corresponding pairs of points (*g_l_* drops to 0 if the solutions are geometrically congruent and the points coincide after rotations and rescaling);
(c) *Canonical correlation* or CANCORR extracts a pair of linear combinations from the U and I coordinate sets, such that the correlation *R*_c_ between them is maximal. It can extract further pairs of linear combinations of coordinates, providing correlations *R*_2_, *R*_3_, *R*_4_ (each new combination being orthogonal to those previously extracted from its respective coordinate set). The number of significant correlations indicates the number of mutually recognizable dimensions shared between the two coordinate sets.

CANCORR is blind to possible differences in the salience of shared dimensions. With this in mind, *Weighted-Euclidean model of individual differences* was used to quantify the effect of inversion, analyzing responses for U and I stimuli in conjunction. This required two “group configurations,” one for each of the MO (female) and WF (male) series, and each based on four (2 subjects × 2 presentation modes) data matrices. Locations of points in these configurations were then held constant, while optimizing the fit to the data by adjusting the dimensional-weight parameters (*w_i_*) for each subject and presentation mode.

## Results

### SDT

**Figure A1:**
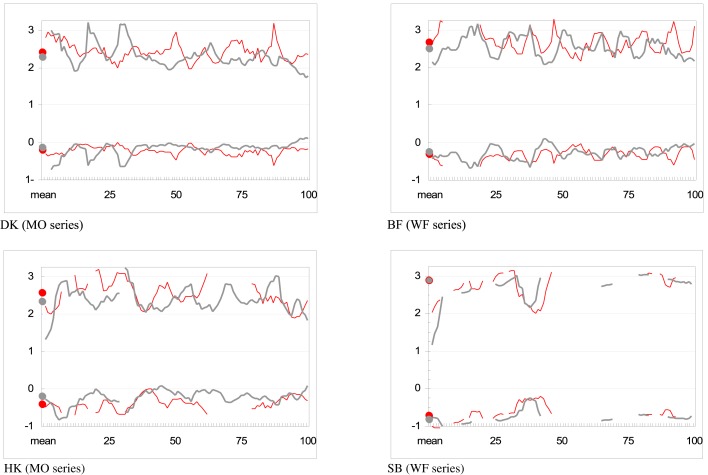
**SDT parameters derived from each subject’s rates of *Same* responses to identical- and different-expression pairs**. Vertical axis: sensitivity measure *d*′ (upper line) and response criterion *C* (lower line); horizontal axis: experimental run. Left-hand points are mean parameters over all 100 runs. 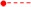 Upright FEs; 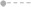 Inverted FEs. Values have been smoothed for clarity, by subjecting the numbers of “hits” and “false alarms” to a running mean with a 5-run window before calculating *d*′ and *C*. Parameters are not defined for runs in which all identical-expression pairs were identified correctly.

The details of the procedure used here mean that only 15/225 = 7% of trials involved physically identical stimuli. However, the subjects frequently gave a *Same* judgment to different-stimulus pairs as well, resulting in *Same* responses for about 25% of the trials. For both presentation modes, the percentage of “false alarms” (different-expression pairs misidentified as *Same*) was highest for subject SB (24.3% of U pairs and 27.3% of I pairs; the difference being significant at *p* = 0.005). In comparison, for DK, the corresponding values were 16.0 and 16.3%; for HK, 19.4 and 17.3% (a significant difference at *p* < 0.001); for BF, 15.8 and 16.0%. This over-vigilance toward “sameness” and willingness to accept false positives is reflected in consistently negative values of the response criterion *C* for all subjects across the course of the experiment (see Figure A1 in Appendix)^3^.

The sensitivity measure *d*′ tends to be slightly higher in the U than in the I mode (Figure A1 in Appendix): that is, different pairs were more distinct from identical pairs when they were presented upright. Plotting *d*′ against experimental run reveals fluctuations from one run to the next, but no obvious evidence of a systematic increase across the course of data collection.

**Figure 2:**
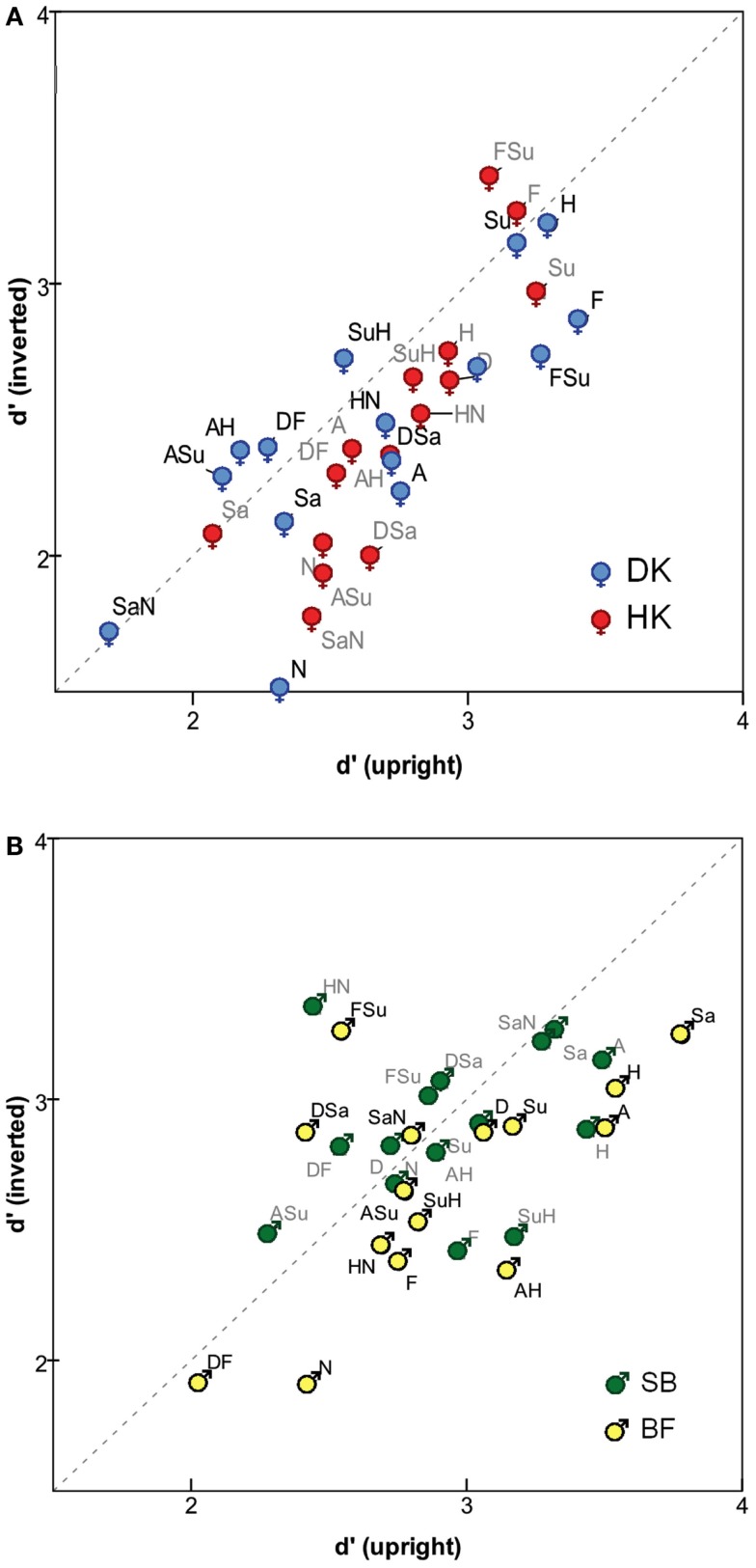
**Sensitivity measure *d*′ for identifying identical pairs of specific expressions, in upright mode (horizontal axis) and inverted mode (vertical axis), for (A) MO series (subjects DK and HK), and (B) WF series (subjects BF and SB)**. Symbols 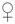 = MO and 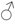 = WF indicate sex of poser, not of observer.

The impact of inversion is also evident when analyzing individual expressions (see Figure 2). Each of the 15 FEs was presented 100 times as an identical-expression pair while 2800 presentations paired it with a different-expression. For each FE, the analysis combines the corresponding rates of *Same* responses. Figure 2 reveals a general trend for inversion to reduce the sensitivity measure *d*′ (more points are below the diagonal), significantly so for three subjects (one-sided Wilcoxon test): *p* = 0.024 for DK, 0.003 for HK, 0.01 for BF, compared to 0.36 for SB.

No consistent inversion effect can be discerned, however, affecting any specific FE prototype or its morphs more than the others. Details among the stimuli vary between the two posers, MO and WF. For the former, *d*′ was highest for *Happiness*, *Surprise*, *Fear*, *FSu*, and *Disgust* (i.e., the difference is relatively easy to detect when these expressions are paired with others) and lowest for *Neutral*, *Sadness*, *SaN*, and *ASu*. For the WF series, *d*′ was highest for *Happiness*, *Surprise*, *Anger*, *Sadness*, and *SaN*, and lowest for *Neutral*, *Fear*, and *ASu*.

**Figure 3:**
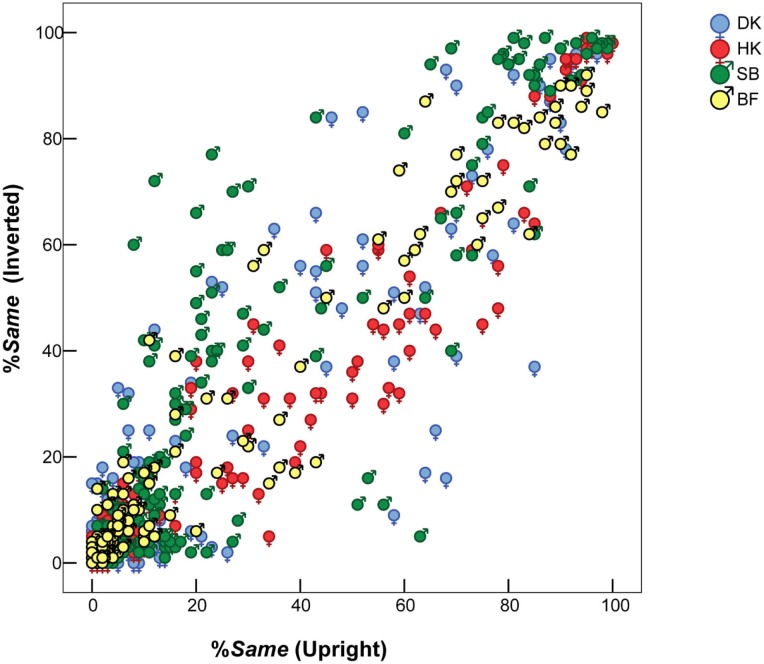
**Similarity between pairs of different FEs – %*Same* responses – when seen upright (horizontal axis) vs. inverted (vertical axis)**. Superimposed results for four observers. Symbols 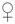 = MO and 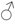 = WF indicate sex of poser, not of observer.

Although the bias toward *Same* responses slightly differed between the two presentation modes, these differences were minor compared to the inter-individual variations. This can be seen in Figure 3, which for individual subjects plots %*Same* responses for each FE pair when seen inverted, against %*Same* for the same pair in the upright mode. A systematic inversion effect would appear as an overall departure from the main diagonal toward the upper left or lower right. Accordingly, HK’s data reveal that he was slightly more likely to judge a given pair of FEs as *Same* when they were *upright* (i.e., inversion of the stimulus pair made any difference slightly more evident to him). Conversely, SB was more likely to judge an *inverted* pair as *Same*.

Figure 3 also shows a concentration of %*Same* values less than 20% from glaringly different FE pairs, but the distribution was not polarized. That is, responses were probabilistic, rather than over-learned and deterministic with a consistent *Same* response for some pairs and a *Different* response for others.

### MDS

### *%Same* matrices: data-set correlations

Correlations *r* between the eight data matrices are tabulated in Table 1. The dominant feature is a distinction between the MO and WF series of expressions, attributable to stylistic variation within an “expression prototype”: posers can differ in the underlying muscle movement involved in an expression, without affecting the emotional message (Bimler and Kirkland, 2001; Alvarado and Jameson, 2002). In addition, even if two posers contract equivalent muscles to express an emotion, they may differ enough in facial structure for these to produce different results.

**Table 1:** **Correlations between individual data matrices**.

	DK-I	HK-U	HK-I	BF-U	BF-I	SB-U	SB-I
DK-U	0.939	0.935	0.930	0.357	0.345	0.313	0.235
DK-I		0.947	0.966	0.382	0.374	0.338	0.252
HK-U			0.977	0.339	0.331	0.299	0.256
HK-I				0.363	0.353	0.330	0.275
BF-U					0.981	0.910	0.803
BF-I						0.929	0.829
SB-U							0.921

*U – upright; I – inverted presentation mode. DK, HK, BF, and SB are individual subjects*.

Table 1 also indicates that if there is any systematic difference between upright and inverted FEs, this is obscured by the larger differences among subjects. For instance, the pattern of judgments for inverted FEs from subject BF is more similar to the pattern for upright FEs from the same subject, than to the pattern for inverted FEs from subject SB.

### Solution dimensionality and variability

We examined separate MDS solutions MO-U, MO-I for the MO (female) images and WF-U, WF-I for the WF (male) images, both when upright and inverted. A common rule-of-thumb for MDS is that the dimensionality of a solution should not exceed *N*/4, where *N* is the number of items (here *N* = 15), but the limit can be relaxed in this situation where multiple data matrices are pooled. The choice of how many dimensions to retain is based on criteria such as the number of *interpretable* axes and the badness-of-fit values (*Stress*_1_). In all four cases (MO-U, MO-I, WF-U, WF-I), four dimensions appeared to be optimal, yielding *Stress*_1_ values of 0.048, 0.039, 0.061, and 0.076. These were substantial improvements on the values for three dimensions (0.080, 0.067, 0.102, and 0.103 respectively).

The robustness of these solutions was demonstrated by comparing them with our earlier four-dimensional MDS solutions for the same posers, MO and WF, derived from sorting-data for upright mode (Bimler and Paramei, 2006). Those solutions included 39 additional morphed items (i.e., 54 stimuli in total), though here we focus only on the coordinates of the 15 items included in the current set.

According to CANCORR, all four dimensions in the present FE spaces have recognizable, independent counterparts in the sorting-data solutions. In particular, in the two MO solutions all four canonical correlations were significant at *p* ≤ 0.002 (χ^2^ test on Wilks’ Λ statistic), inter-item distances are highly correlated (*r* = 0.84), and the locations of points are very similar (Procrustes distance *g*_l_ = 0.034). In the two WF solutions, present and based on sorting-data, all four dimensions again have recognizable counterparts: the four canonical correlations were all significant at *p* ≤ 0.039); inter-item distances are very similar (*r* = 0.78), as are point locations (*g*_l_ = 0.059). This convergence is evidence that the solutions are stable, although each is based on only two data matrices.

### Comparison of solutions for upright vs. inverted facial expressions

Table 2 shows comparisons among these separate solutions, with cophenetic correlations *c* (above the diagonal) and Procrustes distances *g*_l_ (below the diagonal). The high correlation and low *g*_l_ show that very similar spaces represent MO-U and MO-I, and again for WF-U and WF-I. According to CANCORR, all four dimensions of MO-U have recognizable, independent counterparts in MO-I: the smallest canonical correlation is *R*_4_ = 0.988, and all four are significant at (*p* ≤ 0.001, χ^2^ test on Wilks’ Λ statistic). All four dimensions of WF-U have counterparts in WF-I, with canonical correlations ranging down to *R*_4_ = 0.928 (*p* ≤ 0.001). It appears that inversion has had no gross effect on the subjects’ ability to recognize whether two stimuli were the same or different. Given this level of convergence, we omit a detailed scrutiny of the individual solutions.

**Table 2:** **Cophenetic correlations *c* (above the diagonal) and Procrustes distances *g*_l_ (below the diagonal) between 4D MDS solutions, each based on data matrices from two subjects judging FEs of the same poser, MO or WF. U – upright, I – inverted presentation mode; MO – female poser, WF – male poser**.

	MO-U	MO-I	WF-U	WF-I
MO-U	–	0.93	0.78	0.74
MO-I	0.006	–	0.80	0.74
WF-U	0.047	0.051	–	0.95
WF-I	0.051	0.058	0.010	–

### Weighted-Euclidean MDS analysis

A “group configuration” was constructed for both stimulus sets – a compromise or consensus combining four data matrices (2 subjects and 2 presentation modes) – as a pre-requisite for testing whether inversion affects the weight (salience) of the dimensions of FE space. Judging from the *Stress*_1_ values for two, three and four dimensions, a 4D solution was optimal for both modes of presentation and both sets.

**Figure 4:**
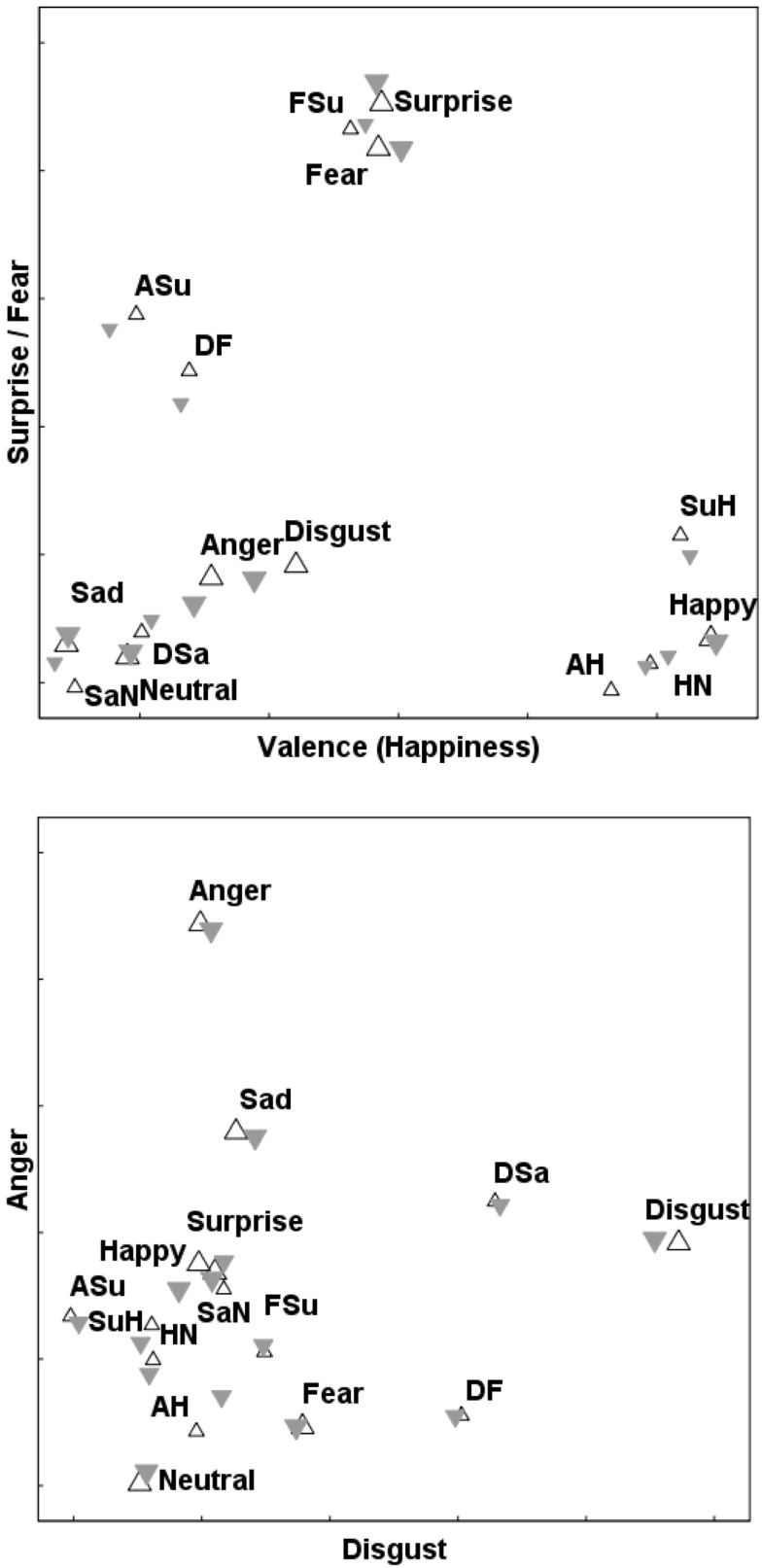
**Superimposed 4D solutions for the upright and inverted FEs of the “MO series” (female poser)**. Projections onto the *D*1/*D*2 (top panel) and *D*3/*D*4 (bottom panel) planes. 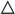 – upright prototypes; 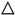 – upright morphs; 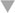 – inverted prototypes; 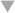 – inverted morphs.

**Figure 5:**
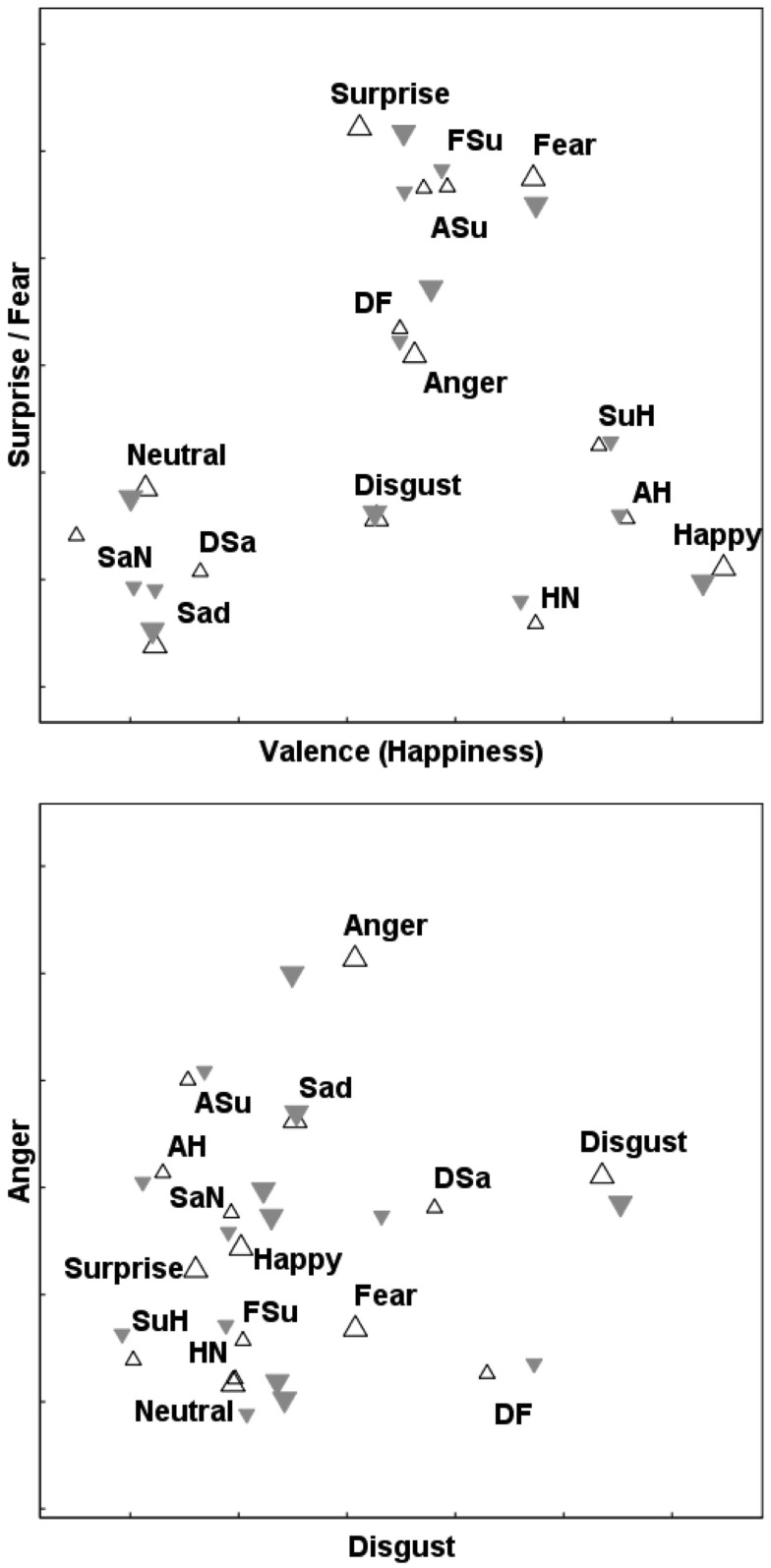
**Superimposed 4D solutions for the upright and inverted FEs of the “WF series” (male poser)**. Projections onto the *D*1/*D*2 (top panel) and *D*3/*D*4 (bottom panel) planes. Symbols as in Figure 4.

This is a convenient point to discuss various features of these maps of FE space. After minor rotation, all four axes lend themselves to straightforward interpretations as continuous affective dimensions. *D*1 is a bipolar “Valence” dimension, running from *Sad* at its negative extreme up to *Happy* (and the part-*Happy* morphs) at the positive extreme. *D*2, *D*3, and *D*4 are unipolar axes of *Surprise*/*Fear*, *Disgust*, and *Anger* respectively. Two views of the MO solution are shown in Figure 4. In the same way, Figure 5 depicts the WF solution.

The dimensional-salience parameters *w_i_* from weighted-Euclidean MDS are listed in Table 3. These optimize the fit between the group configurations, and subjects’ responses under both modes of presentation. DK seems to be more attuned to the *Disgust* axis (*D*3) in upright stimuli; the other subjects do not exhibit this axial difference.

**Table 3:** **Dimensional-salience parameters (*w_i_*) fitting the group configurations to individual subjects’ responses, for the upright and inverted modes of FE presentation**.

Subject/mode	*Happy–Sad*(D1)	*Fear*/*Surprise* (D2)	*Disgust* (D3)	*Anger* (D4)
**MO (FEMALE POSER) SOLUTION**				
DK upright	0.105	0.113	0.144	0.130
DK inverted	0.118	0.119	0.119	0.120
HK upright	0.122	0.116	0.118	0.118
HK inverted	0.120	0.118	0.117	0.120
**WF (MALE POSER) SOLUTION**				
SB upright	0.122	0.120	0.119	0.116
SB inverted	0.115	0.122	0.127	0.120
BF upright	0.118	0.115	0.127	0.123
BF inverted	0.118	0.121	0.120	0.123

### Assessing categorical processing from the MDS solutions

Suggestions of *categorical processing* can be seen in Figures 4 and 5, for stimuli containing any element of *Happiness*. Specifically, the *Surprise*/*Happy* (*SuH*), *Angry*/*Happy* (*AH*), and *Neutral*/*Happy* (*NH*) stimuli are all close to one other and to prototypal *Happiness*, with a gulf between them and the other stimuli. In other words, the perceptual difference between *no* smile and 50% of a smile is considerably greater than the difference between 50% of a smile and a complete smile, implying that even a 50% smile is enough to reach ceiling level on our ability to detect that particular form of mouth curvature as conveying *Happy* emotion.

This impression can be quantified by defining a crude “Categorization Index” (CI). If *d*_1_ is the distance in the MDS solution between a FE morph and one of its “parents,” and *d*_2_ is the distance to the second parent, then CI = *d*_1_/(*d*_1_ + *d*_2_). In the absence of CP, the morph would be located midway between the two prototype-expression points (ignoring the influence of randomness in the data), and CI = 0.5. If the morph is perceptually identical to the first or the second “parent,” then CI reaches its extreme values of 0 or 1 respectively.

**Figure 6:**
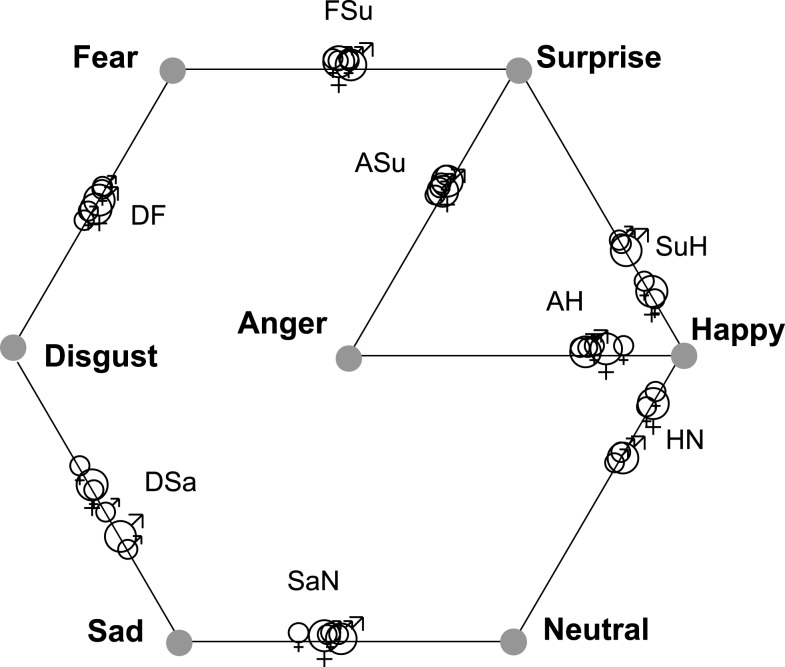
**The symbols on each line show the relative proximity (Categorization Index, CI) of one morph FE stimulus to its two emotion “parents” in 4D solutions**. 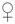, 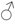 indicate CI for “MO series” (female poser) and “WF series” (male poser) respectively. Smaller symbols indicate CI for either upright or inverted solution, larger for the solution aggregating data for both modes of FE presentation.

Table 4 lists CI for each of the eight morph stimuli, within the combined 4D solutions for each poser (Figures 4 and 5). In addition, CI was calculated for the locations of stimuli in the separate upright and inverted solutions. The CI information presented in Table 4 for these six solutions appears visually in Figure 6, where the six estimates of CI for each morph are shown by the location of symbols along a line between its “parent” stimuli. Figure 6 shows consistent departures from 0.5 for the three *Happiness* morphs. Note that there is no indication that departures are any weaker for the inverted mode. Signs of CP are also apparent for the *Sadness*/*Neutral* (*SaN*) and *Anger*/*Surprise* (*ASu*) morphs, which are consistently displaced in the direction of their respective *Sadness* and *Surprise* “parents.”

**Table 4:** **Categorization Index (CI) values derived from the locations of eight morph stimuli within four-dimensional MDS solutions for the upright and inverted modes of FE presentation**.

Poser	Mode	FSu	DF	DSa	SaN	HN	AH	SuH	ASu
MO (female)	Upright	0.46	0.55	0.50	0.46	0.21	0.74	0.78	0.59
	Inverted	0.51	0.47	0.42	0.36	0.16	0.83	0.84	0.57
	both	0.47	0.52	0.47	0.43	0.19	0.76	0.80	0.58
WF (male)	Upright	0.51	0.55	0.57	0.48	0.36	0.72	0.64	0.56
	Inverted	0.48	0.44	0.70	0.45	0.40	0.70	0.63	0.53
	both	0.51	0.50	0.64	0.48	0.37	0.70	0.65	0.56

*Morphs are 50:50 blends of the following prototype FEs: F – *Fear*; Su – *Surprise*; D – *Disgust*; Sa – *Sadness*; N – *Neutral*; A – *Anger*; H – *Happiness**.

## Discussion

Like McKelvie (1995, p. 327), we began with the expectation “[…] that the effect of inversion would vary with different-expressions because they depend differentially on configural information.” This follows from two assumptions laid out in the Section “Introduction”: first, that inversion selectively impairs the processing of configural relative to featural cues; second, that the relative impact of these types of cues varies from one emotion to another. In particular, we expected the inversion to disrupt discrimination of *Disgust*, a possible example of a “distributed” configuration (Paramei and Benson, 1998; Bimler and Paramei, 2006). If two FE morphs differ by one containing a larger element of (say) *Disgust* than the other, then the expected selective disruption of configural cues should cause this difference to contribute less to pairwise similarity when the pair is inverted (i.e., the difference between them should be less apparent). The inversion effect was also expected for *Surprise*, another candidate for a “configural” expression (Bassili, 1979; Calder et al., 2000).

However, contrary to these expectations, the SDT analysis did not reveal any emotion prototype and its morphs to be more impacted by inversion than the others, moreover showing only an *overall* trend for *d*′ to be slightly lower for inverted than for upright FEs (Figure 2). Reduced discriminability of inverted FEs is in accord with the findings of Narme et al., 2011 (healthy controls data), who conclude that a featural strategy had replaced configural processing, under unlimited exposure. However, those authors additionally found the inversion effect (a significant difference in *d*′ between U and I expressions) to be greater for negative emotions, which, it is argued, require greater perceptual processing resources (Prkachin, 2003). We summarized the %*Same* frequencies with MDS so as to embed the stimuli within “FE space” and examine expressions in the context of the entire emotional gamut. As shown in the Results, none of the three measures employed to compare solutions – cophenetic and canonical correlations, as well as Procrustes distance – revealed substantial differences between the upright and inverted modes of presentation of discriminated FEs. Also the Individual Differences MDS analysis revealed that inversion made no systematic difference to the perceptual salience of any of the emotion prototypes (i.e., the contribution of the corresponding dimension to inter-stimulus similarity). The “maps” showed little difference between upright and inverted presentation, for either poser.

One explanation for the lack of emotion-specific effect from inversion would be that, in spite of the explicit instruction to compare expressed *emotional states*, the data reflect the objective similarity between pairs of FEs as *visual images*, not affective interfaces: it is conceivable that due to the brief exposure of a FE pair, 500 ms, the subjects’ judgments of FE “similarity” only tapped into early stages of visual processing, prior to “the locus of emotion perception.” In particular, low-level processing might treat images as arbitrary patterns of edges and gray tones (Dailey et al., 2002), devoid of affective connotations, and perform the equivalent of pixel-by-pixel comparison [as in the Principal Components Analysis (PCA) treatment of FEs by Calder et al., 2001] or an edge-emphasizing primal sketch (Marr, 1982).

It is also conceivable, though, that similarity judgments in our exposure conditions were based on more advanced visual processing, i.e., featural information. Lipp et al. (2009), employing a visual search task with the *S*/*D* paradigm and an array of nine FEs presented for 6,000 ms – comparable to the time per stimulus available here – found no effect of inversion on either detection speed or verbal ratings. Indeed, Calvo et al. (2012) provide convincing evidence that, at short stimulus duration, *Happy* expressions, both upright and inverted, were identified solely via a smiling mouth, this salient feature making it accessible to the visual system. The authors of both studies conclude that, under conditions that preclude/impair configural processing – as is the case with a short stimulus duration – efficient decoding of expressions is mediated by feature-based information. This accords with the broadly accepted view that featural cues are relatively insensitive to face orientation, compared to the inversion-related impairment of visual processing of configural cues (Maurer et al., 2002; Carbon and Leder, 2005).

It might seem that the close agreement between the model of the “FE space” obtained here and the maps of the cognitive closeness of various emotional concepts (e.g., Russell, 1980), would be enough evidence that the processing of FE stimuli extended beyond early stages and accessed internal semantic representations of emotions and their relationships. However, there are pragmatic reasons to expect a high degree of parallelism between the pattern of objective similarities among FEs, and the pattern of semantic similarities among the corresponding emotions. Under the less-than-ideal conditions of real-world interactions (e.g., Smith and Schyns, 2009), where incomplete or degraded information may cause an observer to misidentify the emotional message of a FE, the consequences of a mistake are minimized if it is at least mistaken for a conceptually related emotion. This parallelism was evident when PCA was applied to a database of digitized FE images, treated purely as vectors of gray tones devoid of any cognitive or affective meaning or dependence on image orientation, and yielded factors that were interpretable as intuitive “expression space” axes (Calder et al., 2001).

This is where the CP phenomenon is pertinent. The morphs were constructed so that the difference between (for instance) the absence of a smile (as in the *Neutral* prototype) and a “half”-smile in the *HN* morph is equivalent to the difference along the same continuum between *HN* and a full smile (the *Happy* prototype). If the geometric FE “maps” accurately reflected physical, objective similarity, then the morphs would be located *midway* between the prototypal “parents.” In fact, the *HN–H* distance proved to be subjectively smaller than the *N–HN* distance; the same applying to the *AH* and the *SuH* morphs. In consequence, the *HN*, *AH*, and *SuH* morphs were all confused relatively often with the *Happiness* prototype, and with one another, and all lie close together in every MDS solution we examined, for both stimulus series (see Figures 4 and 5).

It follows that the subjective difference produced by shifting the proportions of, e.g., *Neutral* and *Happy* depend on the position along that continuum. This non-linear response to varying proportions is a necessary condition of CP (e.g., Calder et al., 1996; Young et al., 1997). As further conditions, the response function should be sigmoidal (and not for instance logarithmic) with the steepest slope at the “Mona Lisa” stimulus, perceived as balanced on the cusp between *Neutral* and *Happy*. To establish these would require data for additional morphs, spaced more closely.

We can only observe that the “Mona Lisa” stimulus contains substantially less than 50% of *Happiness* [cf. the Calvo et al. (2012) blended *Neutral* (upper face)*-Happy* expression]. It is tempting to speak of an expression template attuned to the shape of the mouth – a “smile detector” – which saturates at a ceiling level, at quite mild degrees of curvature, and operates to extract the dominant FE feature at an early stage of FE processing. Notably, abundant evidence has accumulated that *Happy* expressions are processed more rapidly and with greater sensitivity (e.g., Esteves and Öhman, 1993; Leppanen and Hietanen, 2004; Maxwell and Davidson, 2004). Also, recognition of *Happy* prototypes was better than chance for backward-masked exposures as short as ca. 20 ms (Maxwell and Davidson, 2004; Milders et al., 2008). Several studies have demonstrated the visual salience of smiling mouth as the diagnostic feature for recognition of *Happiness* (Smith et al., 2005; Calvo and Nummenmaa, 2008; Schyns et al., 2009; Smith and Schyns, 2009; Calvo et al., 2012).

Other authors have described how heightened discrimination at the “watershed” along a perceptual continuum can arise from general pattern-recognition, feature-integration principles without any special instantiation of “categories” (Ellison and Massaro, 1997; Fiorentini and Viviani, 2009; Calvo et al., 2012), further qualifying the term “CP.” The crucial point is that such models require higher levels of processing. Whether the term applied is “CP” or something else, these non-linear relationships between physical difference and subjective dissimilarities imply that the similarity decisions are indeed accessing some level of semantic processing higher than a basic comparison of gray tones.

Fiorentini and Viviani (2009) found that only discrimination along the Valence axis met all the conditions for CP, i.e., between *Happy* FEs and others – in agreement with the indications of CP for *Happy* FEs demonstrated in the present study. The present data point also to signs of CP along the *Sad–Neutral* (*SaN*) and *Anger–Surprise* (*ASu*) continua, with the *SaN* morph perceived as more similar to *Sadness* and the *ASu* morph as relatively similar to *Surprise*. The emerging categorization of *Happiness* and *Surprise* is in accord with Smith and Schyns (2009) who found that both expressions are low-spatial-frequency rich and involve the mouth as its diagnostic feature more than other prototype FEs.

It is notable that signs of CP along a morphed continuum were found for even shorter exposures, 150 ms, in the *Same*/*Different* task (Suzuki et al., 2004), and in an identification task for prototype emotions, 200 ms (Derntl et al., 2009).

Initial light upon the timescale and hierarchy of FE processing – featural vs. configural distinction; visual vs. affective information – was shed by research recording ERPs. In particular, primary visual processing of a face was found to be defined by the P120/N120, a complex which is too early to be modulated by the emotion category (Campanella et al., 2002). Additionally, the N170 component has been widely regarded as sensitive to faces compared to other objects (Eimer and Holmes, 2002; Ashley et al., 2004). Notably, for inverted faces the N170 component appears later and with lower amplitude. More recent studies report some ERP correlates of emotional content preceding the N170 component, namely a greater frontal positivity around 150 ms (Eimer and Holmes, 2007) and enhanced posterior negativity for *Happy* faces around 130 ms (Schacht and Sommer, 2009). Finally, the emotion-related differences in facial configuration have been linked to ERP components with a peak at around 300 ms (Ashley et al., 2004) and a sustained positive wave after 300 ms (Eimer and Holmes, 2002).

This timescale of FE processing indicated by psychophysiological measures, specifically that the affective content of a FE stimulus is still being processed 300 ms after its onset, can reasonably be linked to the fact that the indications of CP in the present study were as strong for inverted as for upright FEs (Figure 6, Table 3). It seems that the brief (500 ms) exposure of stimulus pairs in this study disrupted face configural processing and, thus, enabled a “snapshot” in the microgenesis of a process of FE analysis (cf. Carbon and Leder, 2005; Schwaninger et al., 2006). The exposure was conceivably too short for processing to reach completion, i.e., the decoding of configural information used for affective discrimination, but sufficiently long to extract low-level visual pattern information and, beyond that, for quick detection of the salient mouth feature that permitted Valence evaluation of FEs.

We speculate that if pairs had been presented longer, the results would have been more dependent on stimulus orientation. This assumption is indirectly supported by the fact that in studies reporting a CP effect for discriminated FEs, typical presentation times were longer, 750 ms (Pollak and Kistler, 2002) or 1000 ms (de Gelder et al., 1997; Roberson et al., 2007). Further, since at longer exposure times (15 s, McKelvie, 1995; unlimited, Derntl et al., 2009) inverting FEs does not completely disrupt their verbal labeling, the latter authors consider that the effect of FE inversion takes the form of a *slowing* of configural processing.

## Conflict of Interest Statement

The authors declare that the research was conducted in the absence of any commercial or financial relationships that could be construed as a potential conflict of interest.

## Footnotes

^1^In addition to accuracy, we also measured response times, RTs, taken to correctly recognize two stimuli as non-identical and used the median RTs for each stimulus pair as a second index of similarity. Our RT analysis reinforces the current conclusions (Paramei et al., 2009), but it exhibits additional features that lie outside the scope of this report.

^2^These two alternatives “involve visual processing of facial stimuli at different levels of increasing perceptual complexity” (Calvo and Nummenmaa, 2008, p. 472). In a more recent account these authors differentiate perceptual, categorical and affective processing stages of FEs (Calvo et al., 2012).

^3^Subject SB, as well as applying the criteria of “sameness” most loosely, also responded more slowly than the other subjects by about 300 ms.

## Appendix

SDT analysis enabled us to address a concern that subjects “over-learned” each stimulus pair during the course of 100 trials (despite the intervals separating the 11 sessions of data collection) and came to provide automatic, stereotyped responses (no longer tapping into the perceptual mechanisms that were the target of the research). However, if responses became over-learned, one would expect sensitivity measure *d*′ to increase with advancing runs. As Figure A1 shows, there is no evidence of this.

Note also that stereotyped responses, if they became the rule at an early stage of data collection (a predictable *Same* or *Different* response for a given pair), would leave no pairs with an intermediate percentage of *Same* responses; but this degree of polarization is not evident in Figure 3.

As an additional precaution we examined individual pairs; the probability of a *Same* response generally remained constant over time: it was *not* a case of an initially stochastic pattern polarizing to deterministic “always *Same*” or “always *Different*” patterns. For sufficiently different pairs where this probability remained at nearly 0 from the start of data collection, and for sufficiently similar pairs where the probability remained at 1, over-learning is irrelevant.

As a final precaution, we obtained four-dimensional MDS solutions for the first half of each subject’s data (i.e., only 50 presentations of each stimulus pair) for comparison with results based on complete-data. In general *Stress*_1_ values were slightly higher for the partial-data solutions (i.e., more random noise) but there was no sign of any systematic changes from excluding the latter 50 trials. For upright stimuli, the cophenetic correlations between complete-data and partial-data solutions were 0.95 for subject BF; 0.95 for SB; 0.96 for DK, and 0.98 for HK. For inverted stimuli the respective correlations were 0.94; 0.97; 0.98; 0.99.
